# New York Letter

**Published:** 1895-06

**Authors:** Ogden C. Ludlow

**Affiliations:** 2309 Seventh Avenue; New York


### *CORRESPONDENCE.*

---

### NEW YORK LETTER.

New York, May 15, 1895.

DIPHTHERIA ANTITOXIN.

As might naturally be expected, the subject of the antitoxin treatment of diphtheria has received a great deal of attention from the medical profession here during the past winter. It has been eagerly tested by numerous physicians, both in hospital and private practice, and its advocacy by one of our leading newspapers has awakened a widespread interest in it among the laity. Again and again, when confronted with a desperate case of croup, or a severe type of nasal or pharyngeal diphtheria, the physician finds himself besieged with anxious inquiries, from the poor and ignorant as well as from the more favored classes, regarding the possibilities of "the new remedy for diphtheria." But to answer the eager questioners intelligently and conscientiously is by no means an easy task, for our experience with the antitoxin treatment is not yet ripe, and all physicians, save enthusiasts, hesitate to speak dogmatically.

A little over a month ago, the Academy of Medicine devoted an entire evening to a consideration of the subject. At this meeting, after the physicians connected with the Board of Heath had presented their experiences and statistics, a general discussion of the subject was participated in by a number of well known practitioners. Doubtless your readers have seen references to this meeting in the various medical journals, for it attracted special attention, not only because of the comparative novelty of the theme, but because of the way in which Dr. Joseph E. Winters so vigorously opposed the treatment. He is not given to assuming any such rôle under ordinary circumstances, and this fact serves to add still greater emphasis to his statements. There has been no end of comment here about it, and some unfortunate personalities have been indulged in. Whatever may be the merits of the controversy, I do not think Dr. Winters' sincerity has been doubted by any one, and the fact that it is know that he visited the Willard Parker Hospital from one to three times daily for a considerable time, in order to thoroughly inform himself regarding this treatment, is a sufficient guarantee that he has not spoken prematurely or thoughtlessly, but has simply given utterance to convictions that have been forced upon him by abundant clinical observation. I have just said "abundant" observation, but it would have been more accurate to say observations as extensive as those of the advocates of this treatment; for all must agree that there has been no opportunity as yet for *abundant* observation of the treatment, and that the presentation of the subject at this meeting was necessarily premature.

Since this much-talked-of discussion on antitoxin, there has been but little said regarding it, yet in a recent meeting of the Pediatric Section of the Academy, in connection with the subject of croup, Dr. A. Caillé stated that he had seen incidentally four cases of croup, in which all the physicians present had expressed the opinion that the only hope for the patients lay in mechanical interference. All four of them had received the antitoxin, and had recovered without any mechanical aid to respiration. The next speaker, Dr. George McNaughton, said that the nineteen cases of laryngeal diphtheria that he had seen in consultation since last December had been treated with antitoxin, and only two of them died. Out of the nineteen cases of laryngeal diphtheria immediately preceding this series, and which had not been treated with antitoxin, thirteen had died. He had tried several different varieties of the serum, and had found them all equally good. In only one instance had he seen anything like a bad result from the antitoxin, and in this case there had been an affection of the joints which certainly did resemble a septic complication. Dr. Joseph E. Winters said that such statistics counted for little in the study of the effects of the treatment of any disease; reliable conclusions could only be drawn from hundreds of observations extending over a considerable time. Thus, Dr. White's first series of eleven intubations at the Willard Parker Hospital had all proved fatal, whereas all of his next eleven intubations recovered. Again, he remembered well seeing at one time a child so desperately ill with croup that it had seemed as if it would hardly survive the night, yet when he called at the child's home to learn why the mother had not reported the condition to him the next morning, he had been astonished to see the child at an open window inhaling the cold, damp air, and with a poultice around its neck and hanging down on its chest. The strangest part of it all was that the laryngeal stenosis had disappeared, and respiration was natural. This was an example, though doubtless an extreme one, of how various diseases were recovered from under the most adverse circumstances, and with little or no medical treatment; yet no sane physician would think of using this case as an argument in favor of treating croup by poulticing the neck and allowing the patient to hang out of a window on a cold, rainy day.

While on the subject of statistics, it may not be amiss to call attention to the fact, that many of the cases now being reported as having been successfully treated with antitoxin have had at the same time the benefit of other and well established methods of treatment, thus detracting very greatly from the scientific value of these records. Again, with all due deference to the ability of Dr. Herman M. Biggs as a scientist, it certainly seems to the writer that any unprejudiced physician listening to the reading of Dr. Biggs's statistics of the antitoxin treatment, could hardly fail to be impressed with one fact, so well expressed in Dr. Winters' opening criticism, viz: that the paper dealt very largely with eliminations.

Those who have read over the foregoing notes, and who have felt with the writer a sense of disappointment and dissatisfaction over the present somewhat dubious position of diphtheria antitoxin, from which every earnest physician hoped so much, may find a little encouragement and relief from what follows concerning the treatment of croup by calomel sublimation. This was the subject of a paper, read by Dr. J. Henry Fruitnight, at the meeting of the Pediatric Section already referred to. The author's experience with this method has extended over more than one hundred cases, so that his utterances should have considerable weight. He said that when the laryngeal stenosis in a given case was observed to be increasing, and was associated with recession of the chest walls, stridulous breathing, and more or less lividity of the surface of the body, the calomel sublimations should be employed. The patient being placed in some form of tent, from five to fifteen grains of calomel are to be sublimed. This could be most conveniently done with the aid of a little instrument manufactured for the purpose by Whitall, Tatum & Company, although any improvised apparatus would answer in an emergency. The sublimations are repeated at intervals of from one-half to three hours, and the treatment may be kept up from one to two days to ten or twelve days. The time that elapses between the fumigations, as also the length of time the patient is kept exposed to the fumes of the burning calomel vary with the severity of the case, but in general, it may be stated that fifteen grains should be sublimed every hour, and the patient should remain in the tent for ten minutes at a time. In reply to the objections that had been made to this treatment, that it produced salivation, diarrhea, marked prostration and profound anemia, the reader of the paper said that he had never seen any untoward results from this treatment except in one instance, and in that case, there was only ptyalism, occurring near the end of a long course of sublimations. Nor had he seen it produce albuminuria in any instance. On three occasions, when mothers had insisted, contrary to his advice, on exposing themselves to the calomel fumes in the tent, notwithstanding that they were pregnant, they had miscarried, so that one should be careful about exposing pregnant women to the calomel. Of course, in many cases intubation would be demanded, but even in these, he felt positive that much better results would follow the practice of continuing the calomel sublimation. The following is a fair example of the cases reported in connection with this treatment: A child of three years was seen at eight o'clock in the morning, and was found to be suffering from severe laryngeal stenosis. It was directed that fifteen grains of calomel should be sublimed every half hour, and that the child should be kept in the tent for ten minutes each time. Returning six hours later with the intention of performing intubation, the physician was agreeably surprised to find that the operation was not required. The child made a good recovery. In conclusion, the author of this paper stated that while the results of these calomel sublimations were often so remarkable as to seem almost incredible to those unacquainted with the method, those who had become familiar with it could not be induced to abandon it for any consideration. It could not be denied that it was purely empirical.

Dr. A. Caillé, in discussing the paper, said that he believed the mercury acted beneficially in these cases both by its local and constitutional effect. It could not be doubted that when the calomel sublimations were begun at a time when there was as yet only a little membranous exudation on the mucous membrane, this remedy was capable of exerting a powerful influence on the disease, probably by its stimulating effect on the glands of the mucous membrane. Dr. George McNaughton, of Brooklyn, said that the treatment of croup by calomel fumigations had originated in his city, and was still a favorite mode of treatment. He then compared the results obtained in the treatment of croup by tracheotomy, intubation and calomel sublimations. Out of 2,417 cases of tracheotomy collected, 586, or 24 per cent., had recovered. Out of 5,546 cases of intubation, 1,691, or 30 per cent., had recovered. Out of 505 cases of croup treated by calomel sublimations, 257, or 54 per cent., had recovered.

So much has been said in this letter about croup, that it is natural to ask ourselves the question: What is croup? Dr. S. H. Dessau replies to this in a recent paper by saying that several conditions, characterized by a croupy cough, have been called "croup," but that in true croup there is an exudation of false membrane on the surface of the larynx; in other words, that it is either primary diphtheria, or pseudo-diphtheria of the larynx. He goes on to show that in a recent report of our board of health, out of 286 reported cases of so-called membranous croup, there was true diphtheria present in eighty per cent., and that in 194 of the cases, the diphtheria originated in the larynx. While it could not be denied that death occasionally occurred as a result of a simple catarrhal laryngitis or "false croup," still this mode of termination is extremely rare. The important points to be observed in differentiating true and membranous croup from false croup or catarrhal laryngitis, are the persistence of the symptoms in true croup, both by day and night, and the insidiousness of its onset. The author's reference to the confusion that had arisen from the use of the term croup, led Dr. Winters to refer to a case that he had recently seen in consultation. The fact that the boy had already been sick a week, and that the stenosis had not been relieved by the insertion of a tube, had led him to auscultate the larynx. Detecting the signs of a foreign body there, the parents were questioned, and it was then learned that the day after the boy had swallowed a piece of egg-shell he had developed the croupy cough. The egg-shell was removed by tracheotomy, and the boy recovered.

2309 *Seventh Avenue.* OGDEN C. LUDLOW, M.D.

